# Active Matrixmetalloproteinase-8 in Periodontal Diagnosis: A Scoping Review

**DOI:** 10.3390/diagnostics15222932

**Published:** 2025-11-20

**Authors:** Lata Goyal, Mehak Gupta, Shubham Sareen, Nur Rahman Ahmad Seno Aji, Vaibhav Sahni, Julie Toby Thomas, Tommi Pätilä, Miika Penttala, Pirjo Pärnänen, Timo Sorsa, Shipra Gupta, Ismo T. Räisänen, Pietro Leone

**Affiliations:** 1 Division of Periodontology, Department of Dentistry, All India Institute of Medical Sciences, Bathinda 151001, India; latagoyal83@gmail.com (L.G.); mehak130199@gmail.com (M.G.); 2 Oral Health Sciences Centre, Post Graduate Institute of Medical Education & Research, Chandigarh 160012, India; shubham725358@gmail.com; 3 Department of Periodontics, Faculty of Dentistry, Universitas Gadjah Mada, Jalan Denta No. 1 Sekip Utara, 10 Sleman, Yogyakarta 55281, Indonesia; nur.aji@helsinki.fi; 4 Department of Oral and Maxillofacial Diseases, Head and Neck Center, University of Helsinki and Helsinki University Hospital, 00290 Helsinki, Finland; drthomastoby@gmail.com (J.T.T.); miika.penttala@helsinki.fi (M.P.); pirjo.parnanen@helsinki.fi (P.P.); timo.sorsa@helsinki.fi (T.S.); ismo.raisanen@helsinki.fi (I.T.R.); 5 All India Institute of Medical Sciences, New Delhi 110029, India; vaibhav.sahni@rcsed.net; 6 Department of Congenital Heart Surgery and Organ Transplantation, New Children’s Hospital, University of Helsinki, 00290 Helsinki, Finland; tommi.patila@hus.fi; 7 Division of Oral Diseases, Department of Dental Medicine, Karolinska Institute, 171 77 Stockholm, Sweden; 8 Private Practice, 80122 Naples, Italy; pietro_leone@hotmail.it

**Keywords:** aMMP-8, activated matrix metalloproteinase-8, matrix metalloproteinase-8, collagenase-2, biomarkers, oral health

## Abstract

**Background/Objectives:** Periodontitis is a chronic inflammatory disease initiated by dysbiotic biofilms and driven by host immune dysregulation, leading to connective tissue destruction and alveolar bone loss. Its early diagnosis is closely linked to the progression and outcome of the disease. Active-matrix metalloproteinase-8 (aMMP-8) is one of the key biomarkers for estimating irreversible collagenolytic degradation. aMMP-8 is among the key biomarkers for estimating irreversible collagenolytic degradation. To evaluate the role of oral fluid/salivary/mouthrinse aMMP-8 tests for early detection and treatment monitoring of periodontitis. **Methods:** A search was conducted in the Medline, Embase, Scopus, and Cochrane databases, restricted to articles published between January 2020 and December 2024 to capture the most recent literature. Pilot, cohort, case–control, and cross-sectional studies were included, while studies not aligning with the objectives of this review, as well as narrative reviews, editorials, and opinions were excluded. **Results:** A total of 276 articles were retrieved. After removing duplicates, 226 studies remained, of which 216 were retained following title, abstract, and keyword screening. From these, 26 studies were finally included in this review. aMMP-8 point-of-care tests appear to be promising tools for early diagnosis, disease severity assessment, and treatment monitoring in periodontitis, though findings remain conflicting across studies. Elevated aMMP-8 levels are associated with active periodontal pathology and tissue destruction, reflecting ongoing collagenolytic degeneration. **Conclusions:** aMMP-8 mouthrinse, saliva, or oral fluid tests can be used for predictive and early diagnosis as well as for treatment monitoring in patients with periodontal disease, and they function effectively as biomarkers for periodontitis.

## 1. Introduction

Periodontal disease develops when harmful bacteria in dental plaque disrupt the normal balance between oral microbes and the body’s immune response. This imbalance triggers an excessive immune-inflammatory reaction in the gum tissues, leading to damage to the supporting structures of the teeth [1,2,3,4,5]. Periodontitis is a chronic disease of an irreversible degenerative nature, characterized by the destruction of both soft and hard tissues of the periodontium [6,7,8,9,10]. Common clinical features accompanying periodontitis include gingival recession, gingival bleeding, deepened periodontal pockets, clinical attachment loss (CAL), and alveolar bone resorption [11,12,13]. At the stage of periodontitis, pathological alveolar bone resorption becomes evident and can be detected by radiographic imaging [14,15]. As a chronic irreversible inflammatory disease, periodontitis is also considered a systemic risk factor [16,17,18,19]. The literature provides substantial evidence supporting relationships between periodontitis and other chronic systemic diseases such as rheumatic and cardiovascular diseases, diabetes, kidney and lung diseases, Alzheimer’s disease, and interstitial lung diseases [20,21,22,23,24,25,26].

Periodontitis is one of the most common diseases worldwide, affecting approximately 11% of the global population [27,28]. Considering these factors, early detection and treatment of periodontitis play a fundamental role in maintaining the general health status of individuals [29,30,31,32]. However, this presents a complex challenge, as the primary obstacle for clinicians is not only to identify the disease itself but rather identifying patients at risk of periodontal disease progression and those who are not [31,33,,34]. Furthermore, discerning the active phases of periodontitis remains a significant challenge [35,36].

As a result, this limitation has prompted medical researchers to explore alternative diagnostic tools, such as biomarkers, which can aid in the early predictive detection of periodontal breakdown [37,38]. Ideally, these biomarkers would also facilitate monitoring of treatment outcomes [39,40,41,42]. One key biomarker is active-matrix metalloproteinase-8 (aMMP-8), also known as collagenase-2, neutrophil collagenase, or polymorphonuclear leukocyte collagenase [43,44,45,46]. This enzyme possesses collagenolytic and proteolytic properties, and its expression in gingival tissues and oral fluids holds significant diagnostic value.

MMP-8 belongs to the matrix metalloproteinase (MMP) family, comprising 26 genetically diverse yet structurally related proteases, of which 24 are found in humans [47,48]. These enzymes are involved in both physiological tissue remodeling and pathological degradation of the extracellular matrix and basement membranes. In addition, MMPs regulate various inflammatory and immune responses [49,50]. In periodontitis, dysbiotic biofilms disrupt host immune homeostasis, triggering a self-perpetuating inflammatory loop that leads to destruction of attachment tissues. Neutrophils play a central role by releasing MMP-8 from their granules, which is subsequently activated to aMMP-8, contributing directly to connective tissue breakdown [51,52,53,54].

**Figure 1:**
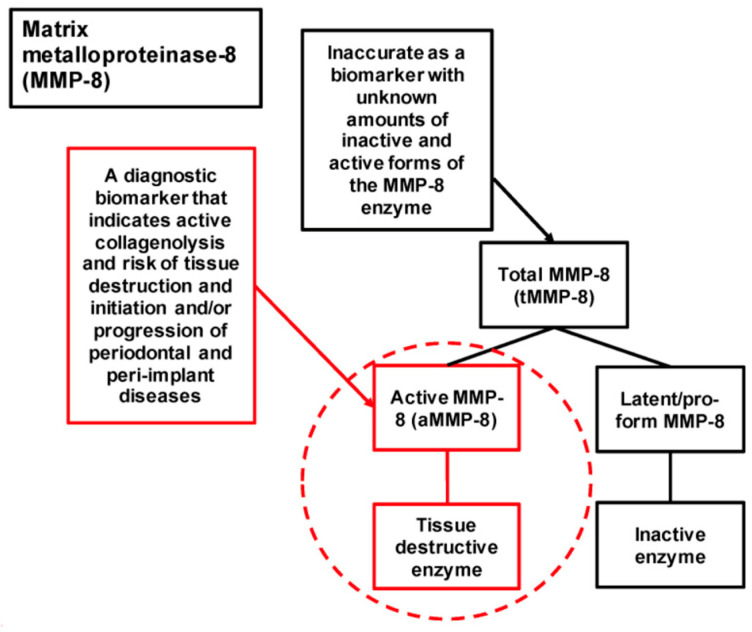
Role of active-matrix metalloproteinase-8 (aMMP-8) as a biomarker and etiological factor in periodontal and peri-implant disease progression, illustrating its utility in identifying at-risk patients and monitoring treatment outcomes (adapted from Räisänen et al., 2023, under Creative Commons Attribution License, CC BY4.0) [63].

MMP-8 can also process bioactive non-matrix substrates such as serpins, complement components, pro- and anti-inflammatory cytokines and chemokines and receptors such as the insulin receptor, thereby modulating immune and cellular responses [55,56]. aMMP-8 appears to be the most prevalent collagenolytic protease expressed in diseased periodontal tissue and oral fluids (saliva, mouthrinse, gingival crevicular fluid [GCF] and peri-implant sulcular fluid [PISF]) [57,58,59]. Studies have shown that MMP-8 is a potential biomarker of periodontal degradation in patients with periodontitis alone and with metabolic diseases like diabetes mellitus or other risk factors like smoking [3,60,61]. aMMP-8 acts both as a key tissue-destructive enzyme and a clinically valuable biomarker, enabling identification of patients at risk for periodontal and peri-implant disease progression and facilitating monitoring of treatment outcomes (Figure 1) [62,63].

**Figure 2:**
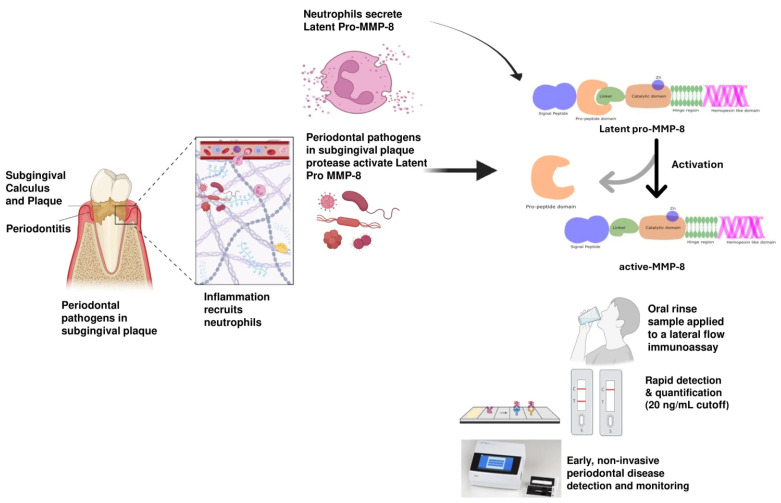
Biological activation pathway of active-matrix metalloproteinase-8 (aMMP-8) in periodontal tissues, and its application in diagnosing and monitoring periodontitis using point-of-care testing. Original image made with BioRender.com. Periodontitis Lesion and Neutrophil Response (left panel) The diagram of the tooth affected by periodontitis with subgingival calculus and dental plaque. These deposits harbor periopathogenic bacteria that trigger an immune response. Neutrophils are recruited into the periodontal pocket and surrounding connective tissue, releasing latent pro-MMP-8. Activation of MMP 8 by Bacterial Proteases (central zoom panel) Within the inflamed periodontal extracellular matrix, bacterial and host proteases cleave the pro-peptide domain of latent MMP-8. This process converts latent pro-MMP-8 into its active collagenolytic form (aMMP-8). The change is depicted as a structural transformation from the intact enzyme with the pro-peptide domain to the active catalytic core bound to zinc (Zn). aMMP-8 as a Collagenolytic Biomarker (text and central arrow): collagenolytic aMMP-8 highlights that this is the biologically active form responsible for collagen breakdown, in contrast to total MMP-8 (which includes inactive forms). Point-of-Care Testing (POCT) Workflow (bottom right sequence) The patient rinses their mouth with a solution to collect GCF-rich mouthrinse (gingival crevicular fluid derivative). The sample is applied to a lateral-flow immunoassay strip, where specific antibodies detect aMMP-8. The test is read visually (two lines = positive, one line = negative) or with a digital reader for quantitative measurement in ng/mL.

Figure 2 illustrates the biological activation pathway of aMMP-8 in periodontal tissues, its role in tissue destruction, and its application in diagnosing and monitoring periodontitis using point-of-care testing.

aMMP-8, with a cutoff value of 20 ng/mL, was introduced as a staging and grading biomarker in the 2017 classification by Sorsa et al. [64]. The primary collagen in the periodontium, type I collagen, is subject to irreversible lysis caused by aMMP-8, alongside the degradation of type III collagen [65,66,67].

Consistently and repeatedly, findings of different studies demonstrate significantly elevated levels of aMMP-8 in oral fluids derived from periodontal tissues exhibiting pathological characteristics [68]. Samples for periodontal biomarker assessment can be obtained from saliva, serum, or mouthrinse, but GCF—collected from the gingival sulcus—offers the highest diagnostic efficacy due to its origin from serum, gingival tissues, and various periodontal cell types. Mouthrinse represents a diagnostically convenient and effective oral fluid, functioning as a practical derivative of GCF for identifying periodontal disease and monitoring treatment outcomes [69,70,71].

With technological advances, various methods for diagnosis have been developed, including lateral flow aMMP-8 point-of-care (POCT) immunoassay tests, such as PerioSafe^®^ and ImplantSafe^®^, used with the ORALyzer© reader. These tests measure the levels of the biomarker aMMP-8 through a fast, simple, non-invasive, and safe technique [72].

MMP-8 has been extensively studied in various biological fluids, including GCF, PISF, saliva, mouthrinse, and serum, for its relevance in periodontitis diagnosis. However, important gaps remain, such as unclear comparative diagnostic accuracy of aMMP-8 across different fluids, variability in methodologies, cut-off values, and populations, along with limited validation of non-invasive fluids and a scarcity of longitudinal studies on disease monitoring. Moreover, few studies have directly compared aMMP-8 with total or latent MMP-8 (tMMP-8) across sample types. Addressing these gaps is crucial to standardize and optimize the clinical utility of aMMP-8 in periodontal disease management.

Given the scattered evidence on the role of aMMP-8 in periodontal disease, a scoping review was chosen to systematically map the available literature, identify key findings, and highlight research gaps. To the best of our knowledge, information regarding the relationship between periodontitis and aMMP-8 has not been systematically reviewed. The objective of this scoping review is to comprehensively map and evaluate the available evidence on the use of aMMP-8 as a diagnostic and prognostic biomarker for the early detection, risk assessment, and monitoring of periodontal disease activity and progression. Additionally, the review aims to compare the diagnostic performance of aMMP-8 with tMMP-8 in various oral fluids.

## 2. Materials and Methods

This present scoping review aimed to address the following research question: What is the role of aMMP-8 as a biomarker in the diagnosis and treatment monitoring of periodontal and peri-implant diseases in adults, as reported in studies published between January 2020 and December 2024?

The comprehensive search was conducted in Pubmed, Scopus, Embase, and Cochrane databases. The search strategy combined terms such as ‘periodontitis’, ‘chronic periodontitis’, ‘gum disease’, ‘aMMP-8’, and ‘active-matrix metalloproteinase-8’ using Boolean operators (AND, OR). Additional studies were identified by hand-searching reference lists of included articles.

**Figure 3:**
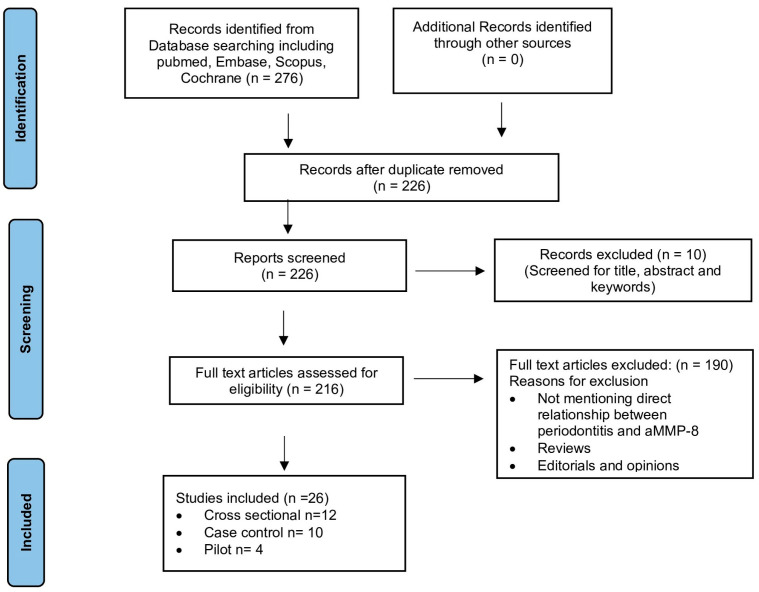
PRISMA-ScR flow diagram.

The last search was performed on 31 December 2024. A PRISMA-ScR flow diagram (Figure 3) illustrates the study selection process. The database search was restricted to the articles of the past 5 years (January 2020–December 2024) to review the recent available literature. Studies were included if they (1) investigated aMMP-8 as a biomarker in periodontitis, (2) were published between January 2020 and December 2024, (3) were cross-sectional, case–control, cohort, or pilot studies, and (4) had full text available in English. Excluded studies included narrative reviews, editorials, opinions, or those not directly addressing the research question. Full-text availability was required to ensure comprehensive data extraction. Data extraction for these studies was conducted to yield information regarding participants, study methodology, results and conclusions. A PRISMA-ScR flow diagram (Figure 3) illustrates the study selection process.

## 3. Results

A total of 276 articles were retrieved from the initial search of PubMed/Medline, Embase, Scopus, and Cochrane databases. After removal of duplicates, 226 articles remained. Screening of titles, abstracts, and keywords eliminated 10 articles. Of the remaining 216 papers, 26 were finally included in the present study after reviewing their full text and applying the inclusion and exclusion criteria.

Two independent reviewers (L.G., M.G.) screened titles and abstracts, followed by full-text review. Disagreements were resolved by a third reviewer to reach a consensus. The selection process adhered to PRISMA-ScR guidelines.

Out of the included studies, twelve were cross-sectional in design, and ten were either cohort or case–control studies, while four were pilot studies. Data were extracted using a standardized form that recorded study characteristics (author, year, location), participant details (number, groups), sample type (e.g., GCF, saliva, mouthrinse), aMMP-8 measurement techniques including POCT, time-resolved immunofluorometric assay (IFMA), and enzyme-linked immunosorbent assay (ELISA), as well as key findings (quantification of aMMP-8 levels, and tMMP-8 levels, sensitivity/specificity), conclusions, and clinical implications.

The summary of the included studies, categorized by design, is presented in Table 1, Table 2 and Table 3. All 26 studies consistently showed elevated aMMP-8 levels in periodontitis and peri-implantitis compared to healthy controls, with GCF and mouthrinse samples demonstrating higher diagnostic accuracy than saliva. The sensitivity of the aMMP-8 POCT kit ranged from approximately 33.2% to 89.7%, while specificity varied between 36.8% and 93.0%, with a cut-off of 20 ng/mL most commonly used for staging and grading (e.g., Sorsa et al., 2020 [64]; Räisänen et al., 2023 [63]).

Studies have also highlighted aMMP-8’s role in monitoring treatment outcomes, with significant reductions observed post-therapy (*p* < 0.05) in cohort studies (e.g., Keskin et al., 2023 [84]). Associations with systemic conditions such as diabetes and rheumatoid arthritis were noted, with aMMP-8 levels correlating with disease severity (e.g., Zhang et al., 2024 [78]).

The Joanna Briggs Institute (JBI) Critical Appraisal Tools were applied according to the study design of each included article. Each study was assessed against relevant checklist domains, including methodological rigor, risk of bias, appropriateness of data collection and analysis, and clarity of reporting (Supplementary Tables S1 and S2). The outcomes of the appraisal were not used as exclusion criteria but were narratively synthesized and considered during the interpretation of findings.

## 4. Discussion

Active-matrix metalloproteinase-8 (aMMP-8) has emerged as a key biomarker for periodontal disease diagnosis due to its enzymatic activity being directly linked to tissue destruction, in contrast to total MMP-8 (tMMP-8), which includes inactive forms. [63,64,71,72,73]. Recent studies indicate that aMMP-8 levels rise predictively during active periodontal tissue destruction involving irreversible collagen breakdown. Although aMMP-8 and tMMP-8 derive from the same gene, the active form offers better specificity and sensitivity for clinical diagnostics [63]. Therefore, it is important not to synchronize the collagenolytic aMMP-8 with non-collagenolytic total, or latentpro-MMP-8 forms in periodontitis and peri-implant diagnostics, as tMMP-8 levels may reflect non-pathological states [62,63,64,66,67,69,73,83].

Relying solely on tMMP-8 may lead to less accurate periodontal assessments. [73,83]. This critical distinction supports the use of aMMP-8 point-of-care testing (POCT) for early diagnosis, treatment monitoring, and confident patient management. However, variability from genetic factors, systemic conditions, and microbial imbalances may affect test reliability across diverse populations. Thus, integrating aMMP-8 measurements with clinical examinations and additional biomarkers provides a more comprehensive disease management approach.

The JBI critical appraisal revealed that most studies adequately addressed confounding factors, though some, like Aji et al. [87] and Guarnieri et al. [88], had unclear strategies for certain confounders, potentially affecting generalizability. The exposure period in case–control studies varied, with some like Keskin et al. and Aji et al. including longitudinal follow-up, while Guarnieri et al.’s study lacked a longitudinal component, limiting causal inference. Overall, the aMMP-8 PoC test emerges as a promising, non-invasive tool for real-time diagnosis and monitoring, although its post-treatment accuracy may be influenced by individual healing patterns, necessitating further research to optimize cut-off values and standardize protocols across diverse populations.

This review underscores aMMP-8 as a valuable biomarker for early periodontal disease diagnosis, supported by numerous cross-sectional, cohort, and case–control studies [69,80,81,82,83,84,85,86,87,88]. Increased aMMP-8 in periodontal patients with metabolic conditions such as diabetes reinforces its role in systemic inflammation [77,79]. Unlike other biomarkers, aMMP-8 actively drives collagenolytic degradation, making the POCT an effective tool for early detection and monitoring of periodontal and related metabolic diseases [53,54,93]. However, influences such as genetics, sample variation, and systemic factors emphasize the need for cautious interpretation and highlight POCT’s role as complementary to clinical evaluation.

aMMP-8 POCT uses oral fluid (mouthrinse) containing active enzyme if periodontal disease is present. Fluid applied to a test strip detects aMMP-8 levels, providing rapid results within 5 min, enabling quick, non-invasive periodontal health monitoring. Studies suggest initial or subclinical periodontitis stages are better detected by aMMP-8 POCT compared to clinical parameters like bleeding on probing [63,79].

Independent studies across various countries have evaluated aMMP-8 POCT diagnostic accuracy using cutoffs of 10, 20, and 25 ng/mL. The 20 ng/mL cutoff is often optimal, demonstrating high sensitivity, specificity, and low false positives. Lower thresholds, such as 10 ng/mL, exhibit high specificity (up to 93.0%) but reduce sensitivity (33.2%), missing periodontitis cases [64,67,69,74,94]. Conversely, studies have shown that adjusting aMMP-8 levels by the number of teeth improves sensitivity (67.1%) with moderate specificity (68.8%) and enhances detection of advanced disease stages (AUROC 0.856). Furthermore, combining aMMP-8 levels with patient factors like age and smoking improves diagnostic accuracy (sensitivity 82.5%, specificity 84.4%), further demonstrating that the test works best as part of a multi-factorial assessment rather than standalone [72,95]. These differences underscore the importance of standardized cutoff values and testing protocols to ensure reliable and reproducible results. Research gaps include performance in peri-implantitis, pediatrics, and across diverse ethnicities.

Beyond periodontal screening, aMMP-8 POCT has been reported to be useful in the screening of diabetes and pre-diabetic/metabolic syndrome patients [77,79,88]. Using alongside questionnaires by healthcare professionals enhances diagnostic accuracy and may facilitate machine learning and AI application development [67].

Wei et al. (2024) report that salivary aMMP-8 POCT predicts periodontitis with moderate accuracy, exhibiting low to moderate sensitivity (around 0.59, 95% CrI: 0.42–0.75) and moderate to high specificity (approximately 0.82, 95% CrI: 0.68–0.93) [94]. Similarly, Li et al. confirm that aMMP-8 POCT provides fair diagnostic accuracy for periodontitis, with sensitivity generally ranging from 0.59 to 0.63 and specificity from 0.82 to 0.84 [95]. These studies emphasize that although elevated aMMP-8 is strongly associated with periodontitis and supports non-invasive risk assessment, the test should not replace a comprehensive clinical examination. However, large independent studies to refine and validate optimal diagnostic cut-offs for broad clinical adoption.

Key methodological differences across studies involve dual assessment combining quantitative aMMP-8 measurement and qualitative visual dipstick evaluation (“–“ or “+”), which improves clarity of borderline results and diagnostic accuracy [63,64,67,69,75,76,79,86,87]. aMMP-8 could be a useful tool for detecting and monitoring periodontal and peri-implant diseases, and combining it with other markers may further improve its diagnostic accuracy, although further research is needed to confirm these benefits [95,96]. Aji et al. (2024) [69] showed that aMMP-8 was found to be a more precise biomarker compared to total MMP-8, MPO, PMN elastase, calprotectin, TIMP-1, and IL-6. This further suggests its role in the proteolytic and oxidative tissue destruction cascade [69]. Additionally, studies among special populations such as head and neck cancer patients confirm that combining aMMP-8 and MMP-9 enhances the prediction of disease progression, underscoring the utility of multi-marker approaches for precision diagnostics [85,97]. Overall, these data support integrating aMMP-8 POCT with complementary biomarkers and clinical factors for more sensitive and specific periodontal disease detection and management.

A recent meta-analysis by Boynes et al. highlighted substantial heterogeneity (I^2^ values exceeding 90%) in salivary tMMP-8 and aMMP-8 studies due to variable designs, saliva collection methods, measurement techniques (e.g., ELISA vs. IFMA), and sample characteristics. Furthermore, funnel plots and Egger’s tests suggest that the reported pooled effect sizes may be overestimated [98]. Evidence shows that aMMP-8 biomarker testing offers significant health economic potential for personalized prevention of periodontal and peri-implant diseases. Although initial testing costs may be higher, implementing aMMP-8 diagnostics as part of secondary prevention strategies can substantially reduce long-term treatment expenses by preventing tooth loss and implant failures [99].

## 5. Limitations

This scoping review’s broad scope limited in-depth quality analysis or meta-analysis, which future systematic reviews should address. A limitation of this review is the predominance of studies conducted by collaborators of the test inventor. Excluding these studies would result in insufficient data to answer the research question, but their dominance may introduce potential authorship bias and limit the generalizability of the findings. Some included studies lacked control for confounding variables such as age, gender, and systemic conditions, potentially affecting biomarker interpretation using oral fluids. Future research should address these factors through rigorous study design and statistical adjustment, ideally in large, longitudinal cohorts with standardized methodologies to enhance validity and generalizability. Although not prospectively registered, this review provides clinically relevant evidence and highlights future research needs.

## 6. Conclusions

aMMP-8 could be a promising biomarker for early detection and monitoring of periodontal and peri-implant diseases, consistently elevated in active disease and systemic conditions such as diabetes. Rapid, non-invasive POCT enables better management but requires integration with clinical evaluation and other markers due to variability and limited validation across populations. Multi-modal diagnostic approaches incorporating aMMP-8 will enable reliable periodontal disease management.

## 7. Future Directions

Integrating aMMP-8 with other biomarkers and clinical parameters in diagnostic frameworks will improve personalized disease management. Combining aMMP-8 with AI-driven models can facilitate early detection, treatment planning, and prevention. Personalized therapy monitoring and real-time digital health tools paired with aMMP-8 POCT hold promise to improve periodontal care outcomes.

## Figures and Tables

**Table 1:** Results on MMP-8 expression in Cross-Sectional Studies.

S.No	Authors/Year/Place of Study	Participants (*N*)/Groups (*n*)	Sample/Technique Used for aMMP-8 and tMMP-8 Quantification	Findings	Clinical Implication
1	Sorsa T et al., 2020, Greece [64]	150 adult participants which include healthy controls and periodontitis.	Mouthrinse/aMMP-8 PerioSafe^®^ test and ORALyzer^®^.	High diagnostic accuracy using aMMP-8 to discriminate healthy vs. periodontitis sites: AUC 0.90 (95% CI 0.83–0.96), *p* < 0.01. Levels classify progression: Grade A < 20 ng/mL, Grade B ≥ 20 ng/mL, Grade C > 30 ng/mL	aMMP-8 cut-off 20 ng/mL effectively distinguishes periodontitis from health, improving early diagnosis and monitoring.
2	Keles Yucel ZP et al., 2020, Turkey [66]	83 (Healthy 23, Gingivitis 20, Stage 3 periodontitis 40)	GCF, serum and saliva/Immunofluorometric assay (IFMA)	Elevated aMMP-8 levels in periodontitis vs. gingivitis and healthy controls aMMP-8 Levels (ng/mL) GCF Healthy: 16.83 ± 9.28 Gingivitis: 44.13 ± 10.6 Peridontitis: 49.42 ± 15.21 Saliva Healthy: 625.74 ± 163.10 Gingivitis: 609.77 ± 174.13 Peridontitis: 779.32 ± 87.26 Serum Healthy: 45.98 ± 39.74 Gingivitis: 106.04 ± 85.27 Periodontitis: 116.05 ± 107.28	MMP-8 is a potential biomarker at both the local and systemic levels for differentiating severe stages of periodontitis.
3	Deng K et al., 2021, Hong Kong [72]	408 (healthy, gingivitis, periodontitis)	Mouthrinse/aMMP-8 PerioSafe^®^ + ORALyzer^®^	aMMP-8 showed a sensitivity of 33.2% and specificity of 93.0% for detecting periodontitis at a cut-off >10 ng/mL. Adjusting aMMP-8 by the number of teeth improved performance with sensitivity 67.1% and specificity 68.8%. The aMMP-8/number of teeth ratio strongly predicted Stage IV periodontitis with sensitivity 89.7%, specificity 73.6%, and AUROC 0.856.	aMMP-8 POCT test has better specificity than sensitivity and is particularly useful for screening or self-assessment rather than ruling out disease.
4	Räisänen IT et al., 2021, Greece [67]	150 patients with periodontitis	Mouthrinse/aMMP-8 POCT lateral-flow immunotest; Saliva/tMMP-8, tMMP-9 ELISA; Saliva/aMMP-9 gelatin zymography	AUC (95% CI) aMMP-8: 0.834 (0.761–0.906) tMMP-8: 0.800 (0.722–0.878) aMMP-9: 0.787 (0.704–0.870) tMMP-9: 0.767 (0.680–0.855)	aMMP-8 offers better screening ability among the tested biomarkers to indicate periodontal tissue breakdown, aiding early detection and monitoring.
5	Öztürk VÖ et al., 2021, Turkey [71]	80 individuals (18 stage III, 19 stage IV periodontitis, 21 gingivitis, 22 health)	GCF, saliva/IFMA; Mouthrinse/aMMP-8 POCT lateral-flow immunotest	aMMP-8 POCT demonstrated Sensitivity 83.9%, specificity 79.2% at 20 ng/mL	aMMP-8 POCT with cut-off 20 ng/mL can be used as non-invasive real-time diagnosis and monitoring tool.
6	Hernandez M et al., 2021, Chile [73]	31 individuals with mild and severe periodontitis.	GCF/ aMMP-8 analysis by IFMA tMMP-8 levels analysis by ELISA.	aMMP-8 demonstrated AUC 0.89 (95% CI 0.83–0.96), sensitivity 98%, specificity 67% at 6.04 ng/mL; tMMP-8 discriminates mild/severe with AUC ≥ 0.80 (95% CI 0.72–0.92), sensitivity 58%, and specificity 96% at a cut-off of 52.79 ng/mL.	aMMP-8 POCT can be a useful adjunct for early diagnosis, severity assessment, and monitoring of periodontitis.
7	Deng K et al., 2022, Hong Kong [74]	95 (61 periodontitis, 34 health/gingivitis)	Unstimulated saliva, oral rinse/lateral flow immunoassay	The aMMP-8 test with cut-off—10 ng/mL on the 1st oral rinse exhibited the best diagnostic accuracy for detecting periodontitis, with a sensitivity of 80.3%, specificity of 67.8%, and an AUROC of 0.740.	The first oral rinse (30 s rinse) provides rapid and reliable discrimination between periodontal health and disease.
8	Gupta S et al., 2023, Greece [75]	150 (periodontitis, gingivitis, healthy)	Mouthrinse, GCF, saliva/aMMP-8 POCT lateral flow, tMMP ELISA	aMMP-8 in oral fluids correlates with clinical parameters. Oral rinse: BOP (r = 0.559), PPD (r = 0.684), CAL (r = 0.770); Saliva: BOP (r = 0.601), PPD (r = 0.705), CAL (r = 0.776); GCF: BOP (r = 0.546), PPD (r = 0.642), CAL (r = 0.743). Elevated aMMP-8 (>20 ng/mL) effectively distinguished periodontitis from health.	aMMP-8 can be a reliable biomarker reflecting tissue inflammation and destruction.
9	Yilmaz D et al., 2024, Turkey [76]	97 (rheumatoid arthritis (RA) periodontitis 26, RA healthy 23, SH periodontitis 24, controls 24)	Serum, saliva/ELISA, immunofluorescence assay	Higher levels of aMMP-8, aMMP-8/TIMP-1 ratio, tMMP-9, MPO, and HNE in periodontitis patients compared to healthy control. Salivary TIMP-1 was more elevated in patients with RA with or without periodontitis. (*p*-value < 0.001 Significant)	aMMP-8 and neutrophil elastase serve as potential biomarkers for both RA and periodontitis.
10	Thomas JT et al., 2025, India [77]	120 (metabolic syndrome with periodontitis 40, healthy with PD 40, healthy 40)	Saliva/aMMP-8 and tMMP-8/ELISA	Levels of aMMP-8, tMMP-8—highest in patients with systemic disease and periodontitis aMMP-8 values (ng/mL) MetS-PD:26.26 ± 3 SH-PD: 24.1 ± 2.56 SH-PH: 14.366 ± 1.89	aMMP-8 could be a potential screening biomarker for periodontitis with metabolic syndrome.
11	Zhang Y et al., 2024, China [78]	80 (healthy 27, periodontitis 29, periodontitis with diabetes 24)	Saliva/ aMMP-8, TNF-alpha, IL-6, IL-8, IL-17 and developmental endothelial locus-1 (Del-1)/ELISA.	aMMP-8 levels were higher in periodontitis (P) and periodontitis with diabetes (PDM) groups compared to the healthy (H) group (*p* < 0.05), with PDM showing higher levels than *p* (*p* < 0.05). aMMP-8 positively correlated with IL-17 (r = 0.77; *p* < 0.01) and negatively correlated with Del-1 (r = −0.69; *p* < 0.01).	Monitoring aMMP-8 levels enables early detection and targeted intervention to prevent progression of both periodontitis and diabetes.
12	Umeizudike KA et al. 2024, Greece [79]	150 individuals with periodontitis and prediabetes	Mouthrinse, saliva/aMMP-8 POCT lateral flow; tMMP-8, elastase, MMP-9/ELISA.	Among stage III grade C periodontitis patients, aMMP-8 levels correlated significantly with prediabetes status (HbA1c ≥ 5.7%) with Spearman’s rho = 0.646, *p* = 0.044. *p*- value—0.001 (Significant)	Elevated aMMP-8 rapidly identifies periodontitis patients with or at risk for prediabetes, enabling integrated management.

**Table 2:** Results on MMP-8 expression in Case–Control/Cohort Studies.

S.No	Authors/Year/Place of Study	Participants (*N*)/Groups *n*	Sample/Technique used for aMMP-8 and tMMP-8 Quantification	Findings	Clinical Implication
1	Raivisto T et al., 2020, Finland [80]	125 participants; gingivitis/ subclinical periodontitis group and healthy controls	Saliva/aMMP-8 by POCT lateral flow immunoassay	Elevated salivary aMMP-8 in 34% of adolescents significantly linked with higher visible plaque index (VPI) % (*p* = 0.005); responsive to treatment with VPI and RC reduction (*p* < 0.05)	Non-surgical therapy reduces aMMP-8; aMMP-8 useful to rule out early inflammation including in orthodontic treatment
2	Mauramo M et al., 2021, Switzerland [81]	202 individuals; controls (86), mild/moderate periodontitis (83), severe periodontitis (33)	Saliva/HLA determination and aMMP-8 IFMA	HLA-A11 allele linked to increased salivary aMMP-8; levels reflect severity and genetic modulation	Measuring aMMP-8 aids personalized risk assessment and targeted management
3	Gupta S et al., 2022, India [82]	102 participants; COVID-19-positive (72), negative (30)	GCF and Mouthrinse/aMMP-8 POCT lateral flow immunoassay	Adjusting for age, gender, and smoking improved test accuracy to 82.35% sensitivity 76.47% specificity (mouthrinse) and 73.53% sensitivity/88.24% specificity (site-specific). AUC from 0.746 to 0.872 (*p* < 0.001) indicates good diagnostic performance.	aMMP-8 POCT useful for screening active periodontal disease in COVID-19 patients
4	Umeizudike KA et al., 2022, UK [83]	189 participants; periodontal health (59), gingivitis (63), periodontitis (67)	Saliva/MMP-8 biosensor, IFMA, ELISA	AUC for differentiating periodontitis and gingivitis from health was 0.81, with diagnostic accuracy of 74.2%. For periodontitis versus health and gingivitis, the AUC was 0.86 with 82.8% diagnostic accuracy.	POCT biosensors and IFMA as effective diagnostic tools to discriminate periodontal disease status and monitor treatment response.
5	Keskin M et al., 2023, Turkey & Finland [84]	52 individuals; controls (25), periodontitis (27)	Mouthrinse/aMMP-8 POCT lateral flow, IFMA, Western immunoblot	The PoC aMMP-8 test demonstrated sensitivity 85.2%, specificity 100%; correlation between aMMP-8 reduction and clinical improvement (*p* < 0.05)	Oral rinse aMMP-8 tests are accurate, responsive, and effective tools for diagnosing and monitoring periodontitis.
6	Brandt E et al., 2023, Turkey [85]	21 periodontitis patients undergoing head and neck radiotherapy	Mouthrinse/PerioSafe^®^, tMMP ELISA	Radiotherapy (RT) for head and neck cancer (HNC) increased aMMP-8 from 21.6 to 54.6 ng/mL (*p* < 0.05) during and after therapy.	aMMP-8, aMMP-9, and IL-6 are potential non-invasive indicators of oral tissue response during cancer therapy.
7	Yilmaz M et al., 2023, Turkey [86]	42 Stage III/IV periodontitis patients	Mouthrinse/POCT, immunofluorescence assay	Sensitivity of the aMMP-8 test reduced from 71.4% baseline to 28.6–42.9% post-treatment; Specificity ranged from 64.3% to 80% at week 6, decreasing to 40–57.1% at week 12, and then slightly increased to 56–64.3% at week 24.	aMMP-8 testing is effective for baseline periodontitis diagnosis, but its accuracy for monitoring treatment outcomes or residual disease declines over time, so clinical assessments must complement biomarker testing.
8	Aji N et al., 2024, Finland [87]	57 participants; Stage III/IV Grade B/C periodontitis (27), healthy (30)	Mouthrinse POCT, gingival tissue/IHC, transcriptomics	IHC demonstrated more Td dentilisin and MMP-8 in CP patients than healthy control. Significant reductions in aMMP-8 were observed after scaling and root planing.	The upregulation of aMMP-8 in response to inflammation makes it a key target for assessing the severity of periodontitis.
9	Aji N et al., 2024, Finland [69]	26 participants; Stage III/IV Grade B/C periodontitis and healthy controls.	Mouthrinse POCT with digital reader; ELISA for tMMP-8	aMMP-8 POCT demonstrated sensitivity 92.3%, specificity 100%; best biomarker with 20 ng/mL cutoff.	aMMP-8 is found to be a precise biomarker for diagnosis and monitoring periodontitis.
10	Guarnieri R et al., 2024, Rome [88]	112 participants; periodontal health, peri-implant health, periodontitis (PER), peri-implantitis (PIM)	GCF, PISF/aMMP-8 POCT mouthrinse with digital reader	Sites with PER and PIM have a higher level of aMMP-8 which positively correlated with other biomarkers aMMP-8 (ng/mL): Periodontal health: 11.58 ± 3.1 Periodontitis: 17.51 ± 9.3 Peri-implant health: 12.42 ± 2.9 Peri-implantitis: 29.8 ± 10.6	aMMP-8 is responsible for destruction of periodontal and peri-implant tissues. Altered neutrophil maturation in periodontitis increases activated neutrophils, driving inflammation and tissue damage.

**Table 3:** Results on MMP-8 expression in Pilot Studies.

S.No	Authors/Year/Place of Study	Participants (*N*)/Groups (*n*)	Sample/Technique used for aMMP-8 and tMMP-8 Quantification	Findings	Clinical Implication
1	Keskin M et al., 2020, Finland [89]	11 head and neck cancer patients	Mouthrinse POCT for aMMP-8. Samples analyzed at 3 points of time (pre-radiotherapy, after 6 weeks of radiotherapy and 1 month after radiotherapy)	Significant changes in aMMP-8 levels with cut-off 20 ng/mL after radiotherapy. aMMP-8 levels (ng/mL) Pre-radiotherapy: 17.99 6 weeks: 75.12 1 month: 38.00	aMMP-8 can be a potential biomarker for identifying periodontal destruction induced by radiotherapy.
2	Kallio E et al., 2024, Finland [90]	112 individuals	Mouthrinse POCT quantified by digital reader.	Patients at increased infection risk had 35.5% higher aMMP-8 values (37.6 ± 49.58) than healthy (27.8 ± 23.3)	aMMP-8 testing is valuable in managing periodontal health in patients undergoing oral surgery, as it enables real-time assessment of inflammation and tissue breakdown.
3	Brandt E et al., 2023, Turkey [91].	13 head and neck cancer patients undergoing radiotherapy	Oral rinse POCT quantified using digital reader	Elevation of aMMP-8 with cut-off 20 ng/mL and fragmented MMP-8 were observed after radiotherapy. aMMP-8 levels (ng/mL) Before radiotherapy: 20.1 3 weeks after radiotherapy: 59.4 1 month after radiotherapy: 38.00	Elevated aMMP-8 in oral rinse can increase the risk of periodontitis after radiotherapy.
4.	Heikkinen et al., 2023, Finland [92]	51 participants with type II diabetes mellitus.	Mouthrinse/aMMP-8 POCT at baseline and post-therapy	At baseline, aMMP-8 levels positively correlated with probing pocket depth 5 mm (r = 0.308, *p* < 0.05), and bleeding on probing (r = 0.298, *p* < 0.05), but not with GHbA1c. Changes in aMMP-8 and GHbA1c during treatment were positively correlated (*p* < 0.01).	The aMMP-8 POCT reliably detects periodontitis and monitors treatment response in type 2 diabetes patients, enabling timely and integrated care.

## Data Availability

No new data were created or analyzed in this study. Data sharing is not applicable to this article.

## Supplementary Materials

The following supporting information can be downloaded at: https://www.mdpi.com/article/10.3390/diagnostics15222932/s1, Table S1: Joanna Briggs Institute (JBI) Critical Appraisal Checklist for Analytical Cross-Sectional Studies. Table S2: Joanna Briggs Institute (JBI) Critical Appraisal Checklist for Case Control Studies.

## Abbreviations

MMP	Matrix metalloproteinase
PoC	Point of care
IFMA	Immunofluorometric assay
