# Supplementary material for: Active Matrixmetalloproteinase-8 in Periodontal Diagnosis: A Scoping Review
Source: Diagnostics (Basel). 2025 Nov 20;15(22):2932. doi: 10.3390/diagnostics15222932 (PMC12651826; doi:10.3390/diagnostics15222932)
Supplement: Supplementary file 1 [file diagnostics-15-02932-s001.zip › diagnostics-3856896-supplementary.pdf]

Supplementary Table S1

Joanna Briggs Institute (JBI) Critical Appraisal Checklist for Analytical Cross-Sectional Studies

Q1	Were the criteria for inclusion in the sample clearly defined?
Q2	Were the study subjects and the setting described in detail?
Q3	Was the exposure measured in a valid and reliable way?
Q4	Were objective, standard criteria used for measurement of the condition?
Q5	Were confounding factors identified?
Q6	Were strategies to deal with confounding factors stated?
Q7	Were the outcomes measured in a valid and reliable way?
Q8	Was appropriate statistical analysis used?

Answers: Yes, No, Unclear, Not Applicable

Cross sectional Studies

No	Cross sectional Studies	Q1	Q2	Q3	Q4	Q5	Q6	Q7	Q8
1	Sorsa T et al., 2020 [64]	Yes	Yes	Yes	Yes	Yes	Yes	Yes	Yes
2	Keles Yucel ZP et al., 2020 [66]	Yes	Yes	Yes	Yes	Yes	Yes	Yes	Yes
3	Deng K et al., 2021 [72]	Yes	Yes	Yes	Yes	Yes	Yes	Yes	Yes
4	Räisänen IT et al., 2021 [67]	Yes	Unclear	Yes	Yes	Yes	Yes	Yes	Yes
5	Öztürk VÖ et al., 2021 [71]	Yes	Yes	Yes	Yes	Unclear	Yes	Yes	Yes
6	Hernandez M et al., 2021 [73]	Yes	Unclear	Yes	Yes	Yes	Yes	Yes	Yes
7	Deng K et al., 2022 [74]	Yes	Yes	Yes	Yes	Yes	No	Yes	Yes
8	Gupta S et al., 2023 [75]	Yes	Yes	Yes	Yes	Yes	Yes	Yes	Yes
9	Yilmaz D et al., 2024 [76]	Yes	Yes	Yes	Yes	Yes	Yes	Yes	Yes
10	Thomas JT et al., 2024 [77]	Yes	Yes	Yes	Yes	Unclear	Unclear	Yes	Yes
11	Zhang Y et al., 2024 [78]	Yes	Yes	Yes	Yes	Yes	Yes	Yes	Yes
12	Umeizudike KA et al. 2024 [79]	Yes	Yes	Yes	Yes	Yes	unclear	Yes	Yes

Supplementary Table S2

Joanna Briggs Institute (JBI) Critical Appraisal Checklist for Case Control Studies

Q1	Were the groups comparable other than the presence of disease in cases or the absence of disease in controls?
Q2	Were cases and controls matched appropriately?
Q3	Were the same criteria used for identification of cases and controls?
Q4	Was exposure measured in a standard, valid and reliable way?
Q5	Was exposure measured in the same way for cases and controls?
Q6	Were confounding factors identified?
Q7	Were strategies to deal with confounding factors stated?
Q8	Were outcomes assessed in a standard, valid and reliable way for cases and controls?
Q9	Was the exposure period of interest long enough to be meaningful?
Q10	Was appropriate statistical analysis used?

Answers: Yes, No, Unclear, Not Applicable

Case Control Studies

No	Case Control Studies	Q1	Q2	Q3	Q4	Q5	Q6	Q7	Q8	Q9	Q10
1	Raivisto T et al., 2020[80]	Yes	No	Unclear	Yes	Unclear	Yes	No	Yes	Yes	Yes
2	Mauramo M et al., 2021 [81]	Yes	No	Yes	Yes	Yes	Yes	Yes	Yes	Unclear	Yes
3	Gupta S et al., 2022 [82]	Unclear	No	Yes	Yes	Yes	Yes	Yes	Yes	Unclear	Yes
4	Umeizudike KA et al., 2022 [83]	Yes	Yes	Yes	Yes	Yes	Yes	Yes	Yes	Yes	Yes
5	Keskin M et al., 2023 [84]	Yes	No	Yes	Yes	Yes	Yes	Yes	Yes	Yes	Yes
6	Brandt E et al., 2023 [85]	Not Applicable	Not Applicable	Not Applicable	Yes	Not Applicable	Yes	Yes	Yes	Yes	Yes

7	Yilmaz M et al., 2023 [86]	Not Applicable	Not Applicable	Not Applicable	Yes	Yes	Yes	Yes	Yes	Yes	Yes
8	Aji N et al., 2024, [87]	unclear	No	Yes	Yes	Yes	No	No	Yes	Not applicable	Yes
9	Aji N et al., 2024[69]	Yes	No	Yes	Yes	Yes	Yes	Yes	Yes	Yes	Yes
10	Guarnieri R et al. 2024 [88]	Yes	No	Yes	Yes	Yes	Yes	Yes	Yes	No	Yes
